# Methodological Challenges in Web-Based Qualitative Research With Medically Underserved Populations

**DOI:** 10.2196/44086

**Published:** 2023-03-30

**Authors:** Alexandra Malia Jackson, Juhee Woo, Marley Olson, Francis Dalisay, Pallav Pokhrel, Clemma J Muller, Scott K Okamoto

**Affiliations:** 1 Public Health Pacific University Forest Grove, OR United States; 2 Department of Sociology Appalachian State University Boone, NC United States; 3 Social Sciences Department Walla Walla Community College Walla Walla, WA United States; 4 College of Liberal Arts & Social Sciences University of Guam Mangilao Guam; 5 Population Sciences in the Pacific Program University of Hawai'i Honolulu, HI United States; 6 Institute for Research and Education to Advance Community Health Washington State University Spokane, WA United States

**Keywords:** data collection, internet, mischievous responders, recruitment, qualitative, technology

## Abstract

Internet- or web-based research is rapidly increasing, offering multiple benefits for researchers. However, various challenges in web-based data collection have been illustrated in prior research, particularly since the onset of the COVID-19 pandemic. To add to the literature on best practices for web-based qualitative data collection, we present 4 case studies in which each research team experienced challenges unique to web-based qualitative research and had to modify their research approaches to preserve data quality or integrity. The first 2 case examples describe issues with using social media to recruit hard-to-reach populations, the third example demonstrates the challenge in engaging adolescents in sensitive conversations on the web, and the final example discusses both the issues in recruitment and the use of different modalities in collecting data to accommodate the medical needs of study participants. Based on these experiences, we provide guidance and future directions for journals and researchers in collecting qualitative data on the web.

## Introduction and Literature Review

The internet has offered a pragmatic, resource-efficient alternative for researchers to access geographically distant or difficult-to-reach populations [1-3]. As the COVID-19 pandemic stopped in-person research and recruitment strategies, researchers shifted to using web-based crowdsourcing recruitment platforms (eg, Amazon MTurk, Prolific, and Qualtrics), recruitment via social media (eg, Twitter, Facebook, Instagram), and use of web-based software for data collection (eg, REDCap, Qualtrics, and Zoom). While necessary during the pandemic, the use of the internet for research also has multiple benefits, including increasing the diversity of research participants and reducing the time and expense of travel. Further, the internet provides more anonymity in participation, which can be essential when collecting sensitive information or data about illegal activities [2,4,5]. As such, internet-based research is likely here to stay.

However, with the increasing use of web-based research and advances in technology, researchers continue to point to challenges in data integrity, particularly in quantitative data collection [4,6-12]. Known issues with fully web-based recruitment and data collection include data farming, bots, mischievous responders (responders who intentionally mislead researchers), low data quality when tasks are completed quickly, and bypassing IP address restrictions (which would allow a participant to complete a survey multiple times) [4,6-12]. To combat the challenges of web-based recruitment and quantitative data collection, researchers have aimed to describe best practices and protocols in survey development, implementation, and data cleaning to promote validity in web-based quantitative data collection [4,6-12].

Similarly, research on conducting synchronous (ie, real-time) web-based interviews and focus groups is growing, mostly since the onset of the COVID-19 pandemic. Research continues to indicate web-based qualitative data collection is viable, offers multiple benefits to researchers, and could be a useful alternative in long term [13-16]. However, similar to quantitative data collection, challenges with the quality of data collected have been reported, including difficulty establishing rapport and managing focus groups on the web, challenges in maintaining or securing privacy, issues with internet connectivity (leading to a loss of data or distraction), and unanticipated distractions during sessions [13,14,17-19]. Additionally, on the part of the participants, unwillingness to participate due to fatigue with video interactions (eg, Zoom fatigue), lack of access to technology, or ability to use technology, has also been reported [13,14,17-19]. While prior researchers have cautioned researchers about fraud, misrepresentation, and mischievous responders (who are frequently identified in web-based quantitative research), these experiences in qualitative data collection have not been discussed in the literature [20].

As collecting synchronous qualitative data on the web (eg, interviews and focus groups) will likely continue, it is imperative that best practices are established to detect and address low-quality or compromised data. We present 4 case studies in which each research team attempted to recruit and engage with a specific population in web-based, synchronous qualitative data collection. Each research team experienced unanticipated challenges in recruiting or collecting qualitative data on the web, requiring changes to recruitment, data collection, or data analytic procedures in their studies. Based on these experiences, we offer additional strategies and best practices for web-based, synchronous qualitative data collection.

### Ethics Approval

Ethical approval was obtained for each project from the following organizations: Pacific University (IRB# 086-22), Appalachian State University (IRB# 22-0112), University of Guam (CHRS# 22-69), and University of Colorado Boulder, (IRB# 19-0684).

## Case Example 1: Native Hawaiian and Pacific Islander Caregivers Study

We aimed to recruit unpaid or family caregivers of Native Hawaiians and Pacific Islanders living with dementia to participate in 2 studies that included web-based quantitative and qualitative data collection. We began recruitment by contacting community-based organizations serving the Native Hawaiians and Pacific Islander community, caregivers, and people living with Alzheimer disease or related dementia. We also posted information about one of the studies on a trial registry website for Alzheimer disease. Because recruiting Native Hawaiians and Pacific Islander adults (a geographically diverse community) via social media was effective for another web-based study [21], we posted a single Facebook advertisement. To screen study participation, we intentionally did not include the link to the survey and asked all interested participants to email the study team. Within 24 hours of posting the advertisement, we received 19 emails with similar content in the subject line and body of the email. We allowed 3 of the 19 participants who contacted us by email to complete the survey. All 3 responders were flagged as bots by the survey software and excluded from further participation. One participant saw information about the study on the Alzheimer disease and dementia trial registry website and reached out to the study team stating they were eligible for the study. That participant completed the survey followed by an interview. On the survey, the participant indicated that they lived on the “West Coast” yet in the interview they said they lived on the “border of Louisiana and Texas.” During the interview, when asked what they appreciated about Native Hawaiians and Pacific Islander culture they stated:

I went to college at a university called [masked]. That's where I met a whole bunch of people and people from everywhere all across the world. I can't tell you how many people there are on a university on the campuses, a lot of people on a campus. I may have been everywhere and I enjoy the culture. Pacific Islander people are very I don't know, I guess, I can say, like he's just so comfortable with himself he's not like he's not shy at all very comfortable just the way that he comes across it comes across as very shy or anything close to being shy.

When asked, “Do you think that there might be anything unique in providing care for the person living with dementia because of their culture?” The participant responded with “um no. I know is to be by side in you know because of his culture if I'm there with him, then I can experience this culture if I'm there with him all the time.” Due to the discrepancy in geographic location reported, the study team decided this participant was likely not a credible participant. As a result, we asked the trial registry to remove the information about the study. To ensure participants were not mischievous responders, we decided to solely recruit participants through snowball sampling and connections with community-based organizations [22]. To screen participants, we asked all interested participants how they heard about the study and checked that the community-based organization was contacted about the study before sharing study materials.

## Case Example 2: Black Women Smokers Study

To examine the role of local context on smoking behaviors among Black women smokers, we recruited women who self-identified as Black, aged 18 years and older, smoked at least 1 cigarette per day for the past 30 days, and lived in the greater Winston-Salem and Greensboro areas in North Carolina. Between the ongoing COVID-19 pandemic and the research team not living in the geographic location participants were recruited from, we initially planned to conduct both in-person and web-based interviews (via Zoom). We first posted flyers on social media (eg, local women’s groups on Facebook) with a link to a survey asking about the eligibility criteria and whether respondents wanted to participate in the study virtually or in person.

On a single day, we received approximately 20 responses to our survey form. Given the large volume of responses on a single day, we checked the time each response was submitted and found that all 20 responses were recorded within a few minutes. This suggested the possibility of 1 person filling out the form with different email addresses. Despite the doubt, we emailed each respondent with further information to schedule an interview. Again, response emails arrived within several minutes of each other with similar content, each requesting a Zoom interview.

We set up a Zoom interview with one of the interested participants. Although turning a camera on during the interview was agreed upon during the recruitment, the interviewee did not turn on her camera, saying her “internet connection is not good.” The interview started, yet her responses were brief about important questions. For example, when asked to describe her neighborhood, the interviewee did not share much other than she lives in “Winston-Salem.” When asked how much she pays for a pack of cigarettes, a question included in the interview protocol to assess the interviewee’s credibility, the interviewee could not answer immediately and said, “about US $20.” Daily smokers are expected to know the price of the cigarettes they smoke. In North Carolina, where cigarettes are cheaper than in other states, smokers can purchase a pack of cigarettes for less than US $10.

Although the recruitment process and several other factors (eg, not turning on the camera and brief answers) already reduced her credibility, we excluded this participant based on her response to the validation question related to the cost of cigarettes. We compensated the participant as advertised and continued receiving multiple emails and survey responses that were likely coming from the same person. As a result, we decided to temporarily stop recruitment for this study and shifted to solely in-person interviews. While we continued web-based recruitment, in-person interviews made it easier for us to assess the credibility of the study participants. First, the interviews were conducted in a public space in Winston-Salem or Greensboro, North Carolina, to prevent individuals who were not actually located in the Winston-Salem or Greensboro area from lying about where they lived. Second, the participants often smoked during the interview, showed their cigarettes, or smelled like cigarettes. Thus, while we continued web-based recruitment, in-person interviews were easier to verify the credibility of the study participants in meeting the eligibility criteria.

## Case Example 3: Culturally Grounded Substance Use Prevention in Guam

To develop a culturally grounded, school-based tobacco and areca nut use prevention curriculum for middle school students in Guam, we recruited Guam middle school students (preadolescents and early adolescents) to participate in small, gender-specific focus groups. This project followed the protocol for formative research involved in developing a prior youth substance use prevention intervention that focused on Native Hawaiian youths [23,24]. With the onset of the pandemic and a restricted timeline, we conducted the focus groups remotely. Participants were recruited with the help of middle school teachers, and all 10 focus groups (*n*_students_=34) were held via Zoom. As an incentive for study participation, we offered participants US $50 gift certificates to a local store. Scheduling the focus groups took far more time than anticipated. Prevention researchers studying school-based samples typically conduct in-person focus groups on campus grounds, right after school. This provides an environment that is convenient and amenable to student engagement in discussions. However, in conducting web-based focus groups, we had limited control over the participants’ environment. In addition, we needed to ensure, and often provide, Wi-Fi access for youth in remote areas with limited resources. This was a logistical challenge that delayed scheduling of the groups. Second, for help with technical assistance or staying engaged in discussions, we sometimes needed to have a parent or guardian who was also willing to be available during the scheduled focus groups. Having a parent present likely affected the data being collected, particularly as we were asking about adolescent substance use. Engagement during the focus groups was also an issue of concern. In the absence of someone in their immediate physical environment to enforce the ground rules, some adolescent participants would walk away during the session, turn off their video, or respond to the moderator’s verbal questions in the chat. The researchers transcribed the discussions with the assistance of a transcription feature on Zoom. However, the chat responses could not be easily incorporated into the transcriptions in the sequence they had occurred during the discussions. Thus, at times, we were unable to identify which interview questions the participants were responding to in the chat. This limited our ability to interpret or make inferences about the data. Lastly, we experienced issues with connectivity and background noise, compromising the quality of the data and the ability to establish a flow or rapport with participants.

Considering these significant barriers to engagement that hindered data quality, we were unable to conduct in-depth or rich text analyses of the youth focus groups. This impacted our ability to examine where Guam youth choose to use or abstain from using tobacco and other substances (eg, home, school, and parks). However, we were able to extract drug-related problem situations, which allowed our team to develop video components for our school-based curriculum.

## Case Example 4: Study of Mild Traumatic Brain Injury Patients

To understand the lived experiences of individuals living with a form of traumatic brain injury (TBI), we conducted web-based interviews with 52 individuals who had experienced one or more traumas in adulthood that resulted in a mild TBI with symptoms persisting longer than 3 months. We recruited participants through snowball sampling and from social media, newsletters, and in-person and internet-based support groups that were familiar to the researcher [22]. To diversify the sample geographically, racially, and economically, we employed paid Facebook advertisements, posted in Facebook groups related to concussion or TBI, Reddit threads related to concussion or TBI, and in the Volunteers sections of Craigslist in major cities across the country. We included information about participant incentives (a US $20 gift card) in all advertisements. Two interviews that were recruited through a Facebook advertisement were excluded due to the participants’ lack of specificity around their diagnosis, treatment plan, and impact of daily functions. One participant initially said her injury was due to a fall but later stated “from when I hit my head on the overhead cabinets.” When asked if she was referring to a different, second incident, she said that she had only had the one. This participant also could not provide information about the medical care she received or how the injury affected her daily life beyond having “like, you know, headaches.” Knowing that the participants we were interested in recruiting often experienced multiple significant symptoms that interfered with daily functioning, we excluded her data. The second participant we excluded was suspicious from the beginning of the interview. When asked basic questions, such as: “How many head injuries have you had?” “When was the most recent head injury?” “What was the diagnosis?” His answers were vague, like “some time ago,” “probably a few,” and “a head injury.” After probing for more concrete answers, he diverted the conversation. Such inconsistencies and lack of specificity reduced the credibility of both these interviewees, leading us to exclude these interviews. We ran a total of 4 Facebook advertisements (posted for 24 hours each) that resulted in 42 participants who indicated interest and then did not participate in the study and 2 participants who were both excluded. Considering the low participation relative to the number of leads collected through these advertisements and the lack of credibility in the 2 interviewees who completed the study, we determined Facebook advertisements were an ineffective mode of recruitment and terminated this type of recruitment effort. Instead, we pursued further recruitment through concussion and TBI-related groups on Facebook. These postings yielded the greatest number of leads and participants. They were also absent of any mischievous responders, likely due to the prescreening processes of becoming a member of those groups. It is worth noting that a significant challenge for this recruitment strategy was the increased time demand and effort. Gaining access to these groups required identifying and requesting permission from the group moderators to advertise the study in their group, and many group moderators denied access to the researchers to their groups.

Due to the symptoms of TBI that include visual deficits, cognitive impairments, and sensory sensitivities, we included participation in interviews using a variety of modes. Participants were able to participate using videoconferencing, phone, and asynchronous data collection via email (both text and audio data) to accommodate their symptoms. The study design, which included a variety of mechanisms for a response, allowed interviewees to participate in the research study in ways that best suited their own needs while minimizing symptom flairs and maximizing participation. Providing an option for individuals to use an audio recorder was important because TBI can affect vision and cognition, making it difficult to write, type, or read. TBIs can also cause cognitive-processing disorders, making it difficult for patients to pull words or express themselves. Asynchronous modalities afforded increased time for interviewees to process the question and formulate their responses. Similar to prior research and Case Example 3, we also faced issues in data collection, including background noise, poor connection, and prematurely terminated interviews despite efforts to reconnect or reschedule with the participants. With these challenges, participants often shifted modalities of participation. For example, several participants whose interviews began on Zoom were finished over the phone due to the participants’ symptoms worsening from the screen time or because the internet connection had been lost. In other cases, we shifted from Zoom to email interviews to provide more time for participants who were developing cognitive fatigue or for participants who were not available to reschedule the interview after experiencing connectivity issues. While this was not our initial study design, the flexibility in a modality of data collection due to the uniqueness of the limited sensory ability to complete a virtual interview, helped to address potential issues with data quality. Had this flexibility not been included, many interviews would have been terminated prematurely, resulting in a loss of data.

## Discussion

All 4 examples illustrate web-based qualitative data collection challenges that are largely missing from the prior literature, yet strongly impact overall data quality. The first 2 case examples describe issues with recruiting specific and hard-to-reach participants through social media and collecting data on the web. In both cases, using social media to reach specific and hard-to-reach populations to complete fully web-based studies led to mischievous or fraudulent participation. While recruitment via social media to reach specific populations may have been fruitful nearly a decade ago [2,3], this web-based method of recruitment and data collection temporarily stopped both projects, hindering the ability to connect with hard-to-reach populations. The third example is slightly different. Rather than issues with recruitment, this example demonstrates the challenge in engaging adolescents in sensitive conversations on the web without physical oversight by the research team. Between the inability to see if participants were physically present, nor could we tell if the responses were compromised due to the technology (eg, responding in the chat rather than verbally), or the presence of a parent or guardian, we were unable to conduct in-depth or rich text analyses of the youth focus groups. While the research team was able to reach and verify participation from their priority population, the team in Case Example 3 faced substantial technical issues in conducting qualitative research on the web, suggesting that web-based data collection with adolescents may not be viable without substantial assistance or alternatives to ensure confidentiality. Similar to the first 2 examples, Case Example 4 highlighted issues in recruitment and participation by mischievous responders. However, the research team found success in recruitment and web-based participation from social media pages that prescreened participants. Additionally, to adapt to the ability of each participant, Case Example 4 illustrated the need to include flexibility and the use of multiple modalities (synchronous and asynchronous) for data collection to engage a priority population (people living with TBI) in web-based research. Based on the lessons learned in these 4 examples, we provide the following recommendations and best practices for web-based qualitative data collection that are consistent with prior research [13,14,17-19].

1. *Including questions in the protocol to verify the identity of the participant.* Ideally, these questions would verify alignment with the inclusion criteria, while building rapport and minimizing harm or stigmatization (eg, the cost of cigarettes, descriptions of diagnoses, or treatment plans). Alternatively, asking demographic questions about the participant using multiple modalities (eg, questions about geographic location in a brief survey and the interview) could aid in identifying mischievous responders.
2. *Using caution when recruiting specific populations via social media.* Consider posting study information on private pages or groups that prescreen participants and not specifying information about compensation. While advertisements can have a substantial reach, they appear to be an easy target for bots and mischievous responders.
3. *Using caution when accessing trial registries for participant recruitment.* While trial registries are an important resource to connect researchers with potential participants, additional eligibility screening may be necessary. Alternatively, trial registries may need to monitor fraudulent behavior, remove study information from publicly available websites, and require potential participants to complete screening measures before accessing information about research studies.
4. *Assessing the efficacy of the modality of web-based data collection with specific populations.* As focus groups are an important method for collecting qualitative data with medically underserved populations [22], creating effective ways to engage adolescents in web-based focus groups is critical. This is evidenced by Case Example 3, where adolescents responded in the chat rather than verbally. Considering adolescents frequently engage in written communication on the web (eg, SMS text messaging and social media), software that supports merging written and verbal qualitative data during focus groups and interviews may be preferable. Allowing asynchronous text participation may also support collecting sensitive information from adolescents. Case Example 4 highlighted the importance of encouraging participation using multiple modalities and the importance of flexibility in the interview process by allowing participants to discontinue remote interviews and submit responses asynchronously to support rich data collection.

Journals need to stay abreast of the challenges of web-based research and encourage transparency from researchers. To better understand these challenges and evaluate the findings, researchers should include a detailed description of recruitment strategies, including explicit prospective criteria (when possible) for excluding mischievous responders. Considering that the number of mischievous or fraudulent participants in research may be increasing, researchers should be encouraged to identify when fraudulent responses are identified and report changes in data collection protocols. Flexibility in the modality of data collection and transparent reports of the data collection process will support rich data collection and evaluation of the findings presented.

### Limitations

The description of these cases has several limitations. First, the separate case studies presented in this article (*N*=4) may not necessarily reflect the experience of other research teams conducting web-based qualitative research methods. Second, researchers’ experiences with web-based qualitative methods could vary by individual-level circumstances, such as levels of institutional information technology infrastructure and support, and by specific research areas and foci. Third, while recruitment of a specific population via social media was hindered by mischievous responders in 2 of the cases presented, this may not be representative of research using social media for the recruitment of a community population. Finally, as technological advances improve, the use of social media evolves, and researchers and research participants become more comfortable with web-based qualitative methods, the challenges presented in this article may be mitigated over time.

### Conclusions

This article describes 4 case studies from geographically dispersed research teams using web-based qualitative methods with medically underserved populations and outlines methodological recommendations and best practices from our experiences. As we describe these best practices, we would be remiss if we did not acknowledge the rapidly changing web-based environment and developing technology. As researchers continue to use web-based methods for qualitative and quantitative data collection, it is essential that journals encourage researchers to acknowledge and discuss their challenges and successful experiences in using technology to collect qualitative data. In addition to losses in data quality, these research teams experienced considerable financial losses including costs to implement the necessary scaffolding to support focus (eg, providing Wi-Fi or requesting parent/guardian support), the cost of advertisements, inefficient researcher time (eg, recruitment or collecting data that were excluded), and compensating mischievous responders. The opportunity to discuss these unanticipated issues in web-based recruitment and qualitative data collection can help to minimize losses to data quality and wasted resources. Thus, the continued identification of best practices is necessary to aid researchers in being more efficient while generating credible data on the web.

## Abbreviations

TBI: Traumatic brain injury
